# Comparative Studies on Polyphenolic Composition, Antioxidant and Diuretic Effects of *Nigella sativa* L. (Black Cumin) and *Nigella damascena* L. (Lady-in-a-Mist) Seeds

**DOI:** 10.3390/molecules20069560

**Published:** 2015-05-26

**Authors:** Claudia-Crina Toma, Neli-Kinga Olah, Laurian Vlase, Cristina Mogoșan, Andrei Mocan

**Affiliations:** 1 Faculty of Medicine, Pharmacy and Dental Medicine, Vasile Goldiș Western University of Arad, 86, Rebreanu Street, Arad 310414, Romania; E-Mails: claudiatoma@uvvg.com (C.-C.T.); neliolah@yahoo.com (N.-K.O.); 2 SC PlantExtrakt SRL, Rădaia 407059, Cluj, Romania; 3 Faculty of Pharmacy, Iuliu Hațieganu University of Medicine and Pharmacy, 8, V. Babes Street, Cluj-Napoca 400010, Romania; E-Mail: mocan.andrei@umfcluj.ro

**Keywords:** *Nigella sativa* L., *N. damascena* L., polyphenols, antioxidants, diuretic, seeds

## Abstract

This study was performed to evaluate the phenolic profile, antioxidant and diuretic effects of black cumin and lady-in-a-mist seeds. In the phenolic profile, differences between the two species are significant. Qualitative and quantitative analyses of the phenolic compounds were performed using a HPLC-UV/MS method. Hyperoside was the only identified flavonoid glycoside (1.08 ± 0.01 μg∙g^−1^ dw plant material), in the *N. damascena* extract. Regarding the flavonol profile, kaempferol was identified before the hydrolysis, only in the *N. sativa* extract (6.06 ± 0.02 μg∙g^−1^ dw plant material) and quercetin only in *N. damascena* seeds (14.35 ± 0.02 μg∙g^−1^ dw plant material). The antioxidant potential of the two species was tested through several electron transfer assays, which indicated, excepting for the FRAP assay, *N. damascena* as exhibiting a higher free radical scavenging activity. The diuretic activity of the two extracts was tested using a rat-experimental model on acute diuresis. Administration of the ethanolic extract of *N. sativa* (100 mg∙kg^−1^) resulted in a significant increase in urine volume, although less than found with the reference drug; in addition *N. damascena* extract did not present a diuretic effect. In reference to the elimination of Na^+^, K^+^ and uric acid, the black cumin extract exhibited a higher natriuretic than kaluretic effect and a similar uricosuric effect with control and *N. damascena*. For *N. damascena*, the Na^+^/K^+^ ratio was sub unitary, but not due to an increasing of the kaluretic effect, but mostly to a decrease of Na^+^ excretion.

## 1. Introduction

Medicinal plants, vegetables and food products are considered to be rich sources of natural compounds that may play an important role in human health, by maintaining it, or preventing and curing diseases. In medicinal plants, these compounds are considered as bioactive natural compounds that can be subsequently developed as new drugs. In foods, they would be defined as phytonutrients, without having therapeutic claims but with important health benefits that can be ultimately used in disease prevention [1,2].

*Nigella sativa* L. (black cumin) is an annual herbaceous plant belonging to the *Ranunculaceae* family, widely grown in the Mediterranean countries, Middle East, Eastern Europe and Western Asia [3]. The black cumin seeds taste hot-peppery and have been used as a spice in several foods, such as bread, yogurt, pickles, sauces and salads [3], but also in the preparation of black cumin paste [4], being considered as a valuable functional food [5]. They are extensively used in traditional medicines in Pakistan, India, China, Saudi Arabia and the countries bordering the Mediterranean region for the treatment of asthma, cough, bronchitis, headache, rheumatism, fever, kidney and liver disorders, influenza, eczema, and as a diuretic, lactagogue, carminative and vermifuge [6]. Recent scientific investigations on *Nigella sativa* seeds and its oil indicate a number of bioactivities for the plant, which include anticarcinogenetic [7], antiulcer [8], antibacterial and antifungal [9,10], antihypertensive [11], hepatoprotective [12], anti-inflammatory, antipyretic and analgesic [13], as well as antioxidant activities such as quenching reactive oxygen species [14], prevention of rheumatoid arthritis in rat models [15], and antihyperlipidemic [16].

*Nigella damanscena* L., commonly known as lady-in-a-mist or ragged lady, is an annual plant belonging to the buttercup family *Ranunculaceae* and widespread throughout temperate regions of Europe [17]. *N. damascena* is grown as an ornamental plant. Its seeds are used in traditional medicine because of their analgesic, anti-edematous and antipyretic effects and, due to their sweet scent of strawberry, to prepare food [18,19].

The two *Nigella* species have been used since ancient times for medicinal purposes but also as a food or preservative for foods [20]. Due to its adaptability, *N. sativa* had a higher commercial value and a larger growing area than *N. damascena* has had, and as a consequence there has also been more investigation into the chemical composition and biological activity of *N. sativa* than that of *N. damascena*. Black cumin produces a wealth of phytochemicals including fixed and volatile oils, proteins, flavonoids, glycosides, alkaloids (mainly magnoflorine), and saponins [21]. *N. damascena* seeds contain fatty and essential oils, proteins, alkaloids, flavonoids and saponins [17,19,21,22].

The full complement of bioactive compounds from *Nigella* species has yet to be elucidated, a step necessary in order to explain their medicinal and alimentary uses. Although *N. sativa* itself is a very competitive subject for phytochemical studies, little is known on the comparative chemical composition of black cumin grown in different regions, and even less on that of other taxa in the genus *Nigella*, in this case, *N. damascena.* However, as one can notice after a literature review, the phytochemical investigations related to *Nigella* species are mostly concerned on volatile or fatty oils composition and their bioactivities and neglect, for instance, their phenolic composition. Dietary phenolic compounds received tremendous attention among nutritionists, food scientists and consumers due to their roles in human health. Research in the past years strongly supports a role for polyphenols in the prevention of degenerative diseases, particularly cancers, cardiovascular and neurodegenerative diseases [23,24]. Also, in recent time, new diuretic drugs have been proposed mainly from natural products and among these, the polyphenolic compounds have received special attention from scientists. Regarding the bioactivities of *N. sativa*, only isolated publications deal with the diuretic activities of essential oil and dichloromethane extract of black cumin’s seeds [25,26], but no data was found related to the *N. damascena* diuretic potential.

To overcome the lack of information regarding the polyphenolic composition and diuretic potential of *N. sativa* and *N. damascena* seeds, the aim of this study was to set a comparative study between these two taxa, in order to bring valuable new data regarding the nutritional value of *Nigella* seeds related to their polyphenolic compounds (phenolic acids, flavonoids and flavonols), antioxidant and diuretic effects. To increase our understanding of the pharmacological and nutraceutical properties of *N. sativa* and *N. damascena* we employed a rapid, highly accurate and sensitive HPLC method in tandem with mass-spectrometry for the simultaneous determination of the phenolic compounds. Furthermore the antioxidant potential of the two species was tested by several assays pointing the different antioxidant facets of *Nigella* extracts. Finally, the diuretic properties of black cumin and lady-in-a-mist seeds were tested using a rat experimental model.

## 2. Results and Discussion

### 2.1. HPLC Analysis of Polyphenolic Compounds

In the first part of these investigations, a target HPLC-UV-MS analysis was carried out to identify nineteen phenolic compounds (caftaric acid, gentisic acid, caffeic acid, chlorogenic acid, *p*-coumaric acid, ferulic acid, sinapic acid, cichoric acid, hyperoside, isoquercitrin, rutin, myricetin, fisetin, quercitrin, quercetin, patuletin, luteolin, kaempferol, and apigenin) in the 70% vol. ethanolic extracts of black cumin and lady-in-a-mist seeds, the results concerning the identified polyphenols being presented in Table 1. In order to obtain more accurate data on flavonoid glycosides and aglycones concentration, and to estimate the nature of hydrolyzed compounds, each sample was analyzed before and after acid hydrolysis.

*p*-Coumaric and ferulic acids were found only in the hydrolyzed extracts. *N. damascena* extract contains the highest amount of *p*-coumaric acid after hydrolysis (4.91 ± 0.02 μg∙g^−1^ dw plant material). The highest value for ferulic acid was registered in the black cumin extract (25.53 ± 0.05 μg∙g^−1^ dw plant material), as seen in Table 1. Compared to the original extracts, the presence of *p-*coumaric and ferulic acid in the hydrolyzed extracts reveals the existence of several glycoside conjugates.

**Table 1:** The polyphenolic compounds content in *N. sativa* and *N. damascena* seeds (μg∙g^−1^ dw plant material).

Polyphenolic Compound	*N. sativa*	*N. damascena*	*N. sativa*_H_	*N. damascena*_H_
*p-*Coumaric acid	NF	NF	4.01 ± 0.03	4.91 ± 0.02
Ferulic acid	NF	NF	25.53 ± 0.05	14.41 ± 0.07
Hyperoside	NF	1.08 ± 0.01	NF	NF
Quercitrin	4.63 ± 0.01	19.58 ± 0.03	NF	NF
Quercetin	NF	14.35 ± 0.02	12.15 ± 0.04	6.925 ± 0.03
Kaempferol	6.06 ± 0.02	NF	105.55 ± 0.12	61.77 ± 0.17

Note: NF—not found, below limit of detection; _H_ hydrolyzed sample. Values are the mean ± SD (*n* = 3).

Regarding the flavonoids pattern, the only identified flavonoid glycoside was hyperoside (1.08 ± 0.01 μg∙g^−1^ dw plant material), in the *N. damascena* extract.

Kaempferol was identified before the hydrolysis, only in the *N. sativa* extract (6.06 ± 0.02 μg∙g^−1^ dw plant material) and quercetin only in *N. damascena* seeds (14.35 ± 0.02 μg∙g^−1^ dw plant material). In the hydrolyzed samples, both flavonols were identified, their highest amounts being registered for *N. sativa* (12.15 ± 0.04 and 105.55 ± 0.12 μg∙g^−1^ dw plant material, for kaempferol and quercetin, respectively).

Data regarding phenolic compounds from these two taxa are quite reduced. Nevertheless, Fico *et al*. identified in the seeds of *N. damascena* 1-*O*-(2,4-dihydroxy)benzoyl glycerol, 3,4-dihydroxy-β-phenethyl alcohol, 2,4-dihydroxyphenylacetic acid, 2,4-dihydroxyphenylacetic acid methyl ester [17] and one year later, 1-*O*-(2,4-dihydroxy)phenylacetyl glycerol [22], but no previous data was found so far regarding the compounds identified in this study.

### 2.2. Determination of Total Phenolic and Flavonoids Content

The results of the total phenolic (TPC) and flavonoids contents in the two analyzed species are represented in Table 2. Thus, the TPC values were expressed as caffeic acid equivalents (mg CAE/g plant material). The calculation of total flavonoid content was carried out using the standard curve of rutin and presented as rutin equivalents (mg·RE/g plant material).

**Table 2:** The total phenolic and flavonoids content.

Samples	TPC (mg CAE/g dw Plant Material)	Flavonoids (mg RE/g dw Plant Material)
*N. sativa*	4.12 ± 0.02	2.01 ± 0.01
*N. damascena*	23.83 ± 2.02	14.53 ± 0.95

Each value is the mean ± SD of three independent measurements; TPC: Total phenolic content; CAE: Caffeic acid equivalents; RE: rutin equivalents.

The extract of *N. damascena* seeds contained the highest amount of total phenolic and flavonoids (23.83 ± 2.02 and 14.53 ± 0.51 mg∙g^−1^ dw respectively), as seen in Table 2. Lower values were measured for the *N. sativa* extract (4.12 ± 0.02 and 2.01 ± 0.01 mg∙g^−1^ dw respectively). Thippeswamy *et al.* found a similar content of total phenolic compounds in an 80% methanolic extract, 4.1 mg galic acid equivalents/g dw by using Soxhlet extraction [14]. Our results are also in line with the findings of Khattak *et al.* who reported similar results for methanolic extracts of black cumin seeds obtained by Soxhlet extraction combined or not with gamma-irradiation [6]. On the other hand, higher values of total phenolic content were reported by Al-Bishri *et al.*, (15.8 mg∙g^−1^ dw) in 95% ethanolic extracts by maceration at room temperature [27]. Regarding the total flavonoid content of *N. sativa*, Ratz-Lyko *et al.* reported a significantly higher content of 19.25 mg∙g^−1^ dw quercetin equivalents in a seedcake 50% ethanolic extract [9]. No previous data regarding the total phenolic and flavonoids compounds in *N. damascena* were found.

### 2.3. Antioxidant Activity

Free radicals are extremely reactive species and are known to damage proteins, to cause breakdown of DNA strands, initiate the peroxidation of various compounds and thus lead to many health problems and degenerative diseases such as cancer, atherosclerosis or inflammation and accelerated ageing. Plants are well recognized for their potential antioxidants constituents, which include flavonoids, tannins, lignin, *etc*. These compounds therefore play a significant role in health promotion [6].

The antioxidant activity of the ethanolic extracts of *N. sativa* and *N. damascena* seeds was evaluated using the DPPH bleaching assay, Trolox equivalent antioxidant capacity (TEAC) method, Ferric ion reducing antioxidant power assay (FRAP) and cupric ion reducing antioxidant capacity (CUPRAC), the results being summarized in Table 3.

**Table 3:** Antioxidant capacity parameters obtained using several methods for studied samples.

Samples	DPPH IC_50_ (µg∙mL^−1^)	TEAC IC_50_ (µg∙mL^−1^)	FRAP (μM TE/g)	CUPRAC (μM TE/g)
*N. sativa* seeds	624.7 ± 12.77 ^a^	77.7 ± 1.26 ^a^	7.45 ± 0.33 ^a^	13.7 ± 0.54 ^a^
*N. damascena* seeds	177.6 ± 3.71 ^b^	31 ± 0.26 ^b^	2.1 ± 0.03 ^b^	26.1 ± 1.01 ^b^
Trolox	50.4 ± 0.21 ^c^	17.4 ± 0.18 ^c^	-	-

Notes: Each value is the mean ± SD of three independent measurements; TE: Trolox equivalents. Results marked with different letters showed highly significant statistical difference (*p* < 0.001).

In the DPPH assay, the antioxidant activity of the extracts was compared with a positive control of Trolox solution. The highest radical scavenging activity was shown by *N. damascena* with a IC_50_ = 177.6 ± 3.71 µg∙mL^−1^ followed by black cumin’s seeds which exhibited a 3.5 time less antioxidant activity (IC_50_ = 624.7 ± 12.77 µg∙mL^−1^). Similar results, regarding *N. sativa* seeds were found also by Khattak *et al.* by testing methanolic extracts [6]. Ratz-Lyko *et al.* reported an IC_50_ of 500 µg∙mL^−1^ and 350 µg∙mL^−1^ for *N. sativa* seeds, before and after hydrolysis in a 50% ethanolic extract [9]. As already proved by Mousa *et al.*, high concentrations of flavonoids and flavonols are reflected in the significant scavenging properties [28]. Higher results were also reported by Thippeswamy *et al*., (IC_50_ = 1.24 mg/dw) in an 80% methanolic extract [14].

The TEAC results are in agreement with the DPPH values, showing that *N. damascena* seeds exhibit a significantly higher antioxidant effect. Higher values for the IC_50_ (IC_50_ = 475 µg∙mL^−1^) were reported by Ratz-Lyco *et al*., in a 50% aqueous-ethanolic extract, suggesting lower antioxidant activity [29]. Concerning the ferric reducing ability, there were some changes in the ranking order, the black cumin seeds presented a better antioxidant capacity than the lady-in-a-mist seeds. Antioxidant investigations by using FRAP method were also carried out by Șen *et al*. who reported a dose-dependent free radical scavenging capacity for a 70% aqueous-methanolic extract [30].

Reports that describe the use of CUPRAC method for the investigation of natural extracts antioxidant potential are quite limited [31]. In this case, the results from the CUPRAC assay are correlated with both DPPH and TEAC assays and indicate *N. damascena* as having a superior antioxidant activity (26.1 ± 1.01 μM TE/g).

The antioxidant activity of *N. sativa* and *N. damascena* seeds was tested by using four electron-transfer based antioxidant assays. The results of the DPPH, TEAC and CUPRAC methods were in agreement also with the total phenolic and flavonoids content, indicating *N. damascena* seeds as having a significant higher antioxidant activity.

### 2.4. Evaluation of Diuretic Activity of N. sativa and N. damascena Extracts

Diuretics modulate the volume and composition of body fluids in a variety of clinical conditions such as hypertension, heart failure, nephritic syndrome and cirrhosis. Herbal diuretics produce very little toxicity and are considered important alternatives that have greater effectiveness and fewer side effects [32]. Many of the herbs used in folk medicine have yet to be scientifically evaluated for their effectiveness and safety. Scientific reports regarding the diuretic effects of *Nigella* species are far limited or even absent. Therefore, it is mandatory to bring new data regarding the influence of these medicinal plants with nutritional values on diuresis process.

#### 2.4.1. Effect on Urine Volume and Uric Acid Excretion

The reference diuretic (furosemide) significantly increased the urine output compared to control (*p* < 0.001), with a diuretic index of 4.25, as seen in Table 4. Administration of the ethanolic extract of *N. sativa* (100 mg∙kg^−1^) also resulted in a significant increase (0.001 < *p* < 0.05) in urine volume, although less than found with the reference drug. However, the *N. damascena* extract did not show any significant modification on the diuresis process, compared to control. The diuretic indexes for these two extracts were 1.53 and 1.01, respectively, compared to 4.25 found for furosemide.

**Table 4:** Effect of *Nigella* ethanolic extracts and furosemide on urine output and uric acid in 24 h of urine collection. Drugs were given orally (*n* = 6).

Group	Dose	Diurese (mL∙kg^−1^/24 h)	Diuretic Index	Uric Acid (mg∙kg^−1^/24 h)
Control		9.54 ± 0.93	1	2.7 ± 0.26
Furosemide	30 mg∙kg^−1^	40.58 ± 6.39 ^A^	4.25	2.55 ± 0.43
*N. sativa*	100 mg∙kg^−1^	14.59 ± 2.85 ^a^	1.53	2.32 ± 0.45
*N. damascena*	100 mg∙kg^−1^	9.61 ± 1.47	1.01	2.35 ± 0.36

Notes: Each value is the mean ± SD of three independent measurements; Diuretic index (DI) = volume treated group/volume control group; Results marked with capital letters showed highly significant statistical difference (*p* < 0.001) compared to control; results marked with uncapitalized showed significant statistical difference (0.001 < *p* < 0.05).

The diuretic effect of the orally administered ethanolic extracts of *N. sativa* and *N. damascena* was evaluated in normal adult male Wistar rats and compared with that produced by furosemide, a loop diuretic widely used in clinical practice. Referencing to the uric acid elimination, no significant difference was registered between the control and the treated groups, for 24 h (*p* > 0.05).

#### 2.4.2. Effect on Urinary Electrolytes Excretion

Diuresis has two components: an increase in urine volume (water secretion) and a net loss of solutes (*i.e.*, electrolytes) in the urine. These processes may result from suppression of renal tubular reabsorption of water and electrolytes into the blood stream [33]. This study put forth the diuretic potential of *N. sativa* and *N. damascena* 70% ethanolic extracts. The electrolyte excretion after oral administration of the ethanolic extracts is shown in Table 5. The administration of furosemide (30 mg∙kg^−1^), as a reference diuretic, led to a significant increase in electrolyte excretion with 4.41 ± 0.69 mM/kg/24 h Na^+^ and 3.61 ± 0.56 mM/kg/24 h K^+^ (Table 5) (0.001 < *p* < 0.05).

**Table 5:** Effect of *Nigella* ethanolic extracts and furosemide on the concentrations of urinary electrolytes in 24 h of urine collection. Drugs were given orally (*n* = 6).

**Group**	**Dose**	**Na^+^ (mM/kg/24 h)**	**K^+^ (mM/kg/24 h)**	**Na^+^/K^+^ Ratio**	**Saluretic Index**	
					Na^+^	K^+^
Control		2.74 ± 0.26	2.13 ± 0.21	1.28	1	1
Furosemide	30 mg∙kg^−1^	4.41 ± 0.69 ^a^	3.61 ± 0.56 ^a^	1.22	1.60	1.69
*N. sativa*	100 mg∙kg^−1^	3.23 ± 0.63	2.84 ± 0.55	1.13	1.17	1.33
*N. damascena*	100 mg∙kg^−1^	1.4 ± 0.21 ^a^	2.12 ± 0.32	0.66	0.51	0.99

Notes: Each value is the mean ± SD of three independent measurements; Saluretic index (SI) = (mM treated group)/(mM control group); Results marked with uncapitalized letters showed significant statistical difference (0.001 < *p* < 0.05).

After the administration of *N. sativa* extract an increase in the electrolytes excretion was registered, however this was not statistically significant, as seen in Table 5. Nevertheless, the above par natriuretic ratio indicates a favorable natriuretic activity of *N. sativa* extract [32]. Regarding *N. damascena* extract, a significant decrease in sodium excretion was found correlated also with a sub unitary natriuretic ratio. However, no modification regarding the potassium excretion was registered, compared to control.

All in all, in the present study, in reference to the elimination of Na^+^, K^+^ and uric acid, the extract of *N. sativa* showed a greater natriuretic than kaluretic effect and a similar uricosuric effect with control or *N. damascena*. The Na^+^/K^+^ ratio can predict the nature of the diuretic mechanism. For *N. damascena*, the Na^+^/K^+^ was sub unitary, but not due to an increasing of the kaluretic effect but mostly to a decrease of Na^+^ excretion.

## 3. Experimental Section

### 3.1. Plant Materials and Extraction Procedure

The vegetal material from *N. sativa* and *N. damascena* seeds was harvested from Arad County, Romania, in the summer of 2014. Voucher specimens (Voucher Nos. 10/2014 and 11/2014) were deposited in the Herbarium of the Pharmacognosy laboratory of the Vasile Goldiş Western University of Arad, Romania. The plant material was air dried at room temperature in the shade, separated and ground to a fine (≤300 µm) powder and then extracted. One gram of each sample was weighed and macerated with 5 mL of 70% ethanol, for 10 days. The samples were then centrifuged at 4000 rpm for 30 min, and then the supernatant was recovered. In order to obtain more accurate data on flavonoid glycosides and aglycones concentration, each sample was analyzed before and after acid hydrolysis. Extractive solution (2 mL) was treated with 2 M hydrochloric acid (2 mL) and ascorbic acid solution (0.2 mL, 100 mg∙mL^−1^), and the mixtures were heated at 80 °C on a water bath for 30 min, ultrasonicated for 15 min, and heated for another 30 min at 80 °C. During the heating, methanol (1 mL) was added to the extraction mixture every 10 min, in order to ensure the permanent presence of methanol. The mixtures were centrifuged at 4000 rpm and the solutions were diluted with distilled water in a 10 mL volumetric flask and filtered through a 0.45 μm filter before injection.

### 3.2. Chemical and Instrumentation

Chlorogenic acid, *p*-coumaric acid, caffeic acid, rutin, apigenin, quercetin, isoquercitrin, quercitrin, hyperoside, kaempferol, myricetin, fisetin from Sigma (St. Louis, MO, USA), ferulic acid, sinapic acid, gentisic acid, patuletin, luteolin from Roth (Karlsruhe, Germany), cichoric acid, caftaric acid were from Dalton (Toronto, ON, Canada). HPLC grade methanol, ethanol, hydrochloric acid, ferric chloride, copper chloride and Folin-Ciocalteu reagent were purchased from Merck (Darmstadt, Germany), while 2,4,6-tris(2-pyridyl)-s-triazine radical (TPTZ), 2,2′-azinobis-3-ethylbenzothiazoline-6-sulphonic acid (ABTS), 2,9-dimethyl-1,10-phenantroline (Neocuproine), sodium molybdate dihydrate, sodium nitrite, sodium hydroxide, sodium carbonate, sodium acetate trihydrate, and anhydrous aluminum chloride were from Sigma-Aldrich (Steinheim, Germany). 2,2-Diphenyl-1-picrylhydrazyl (DPPH) and 6-hydroxy-2,5,7,8-tetramethylchroman-2-carboxylic acid (Trolox) were obtained from Alfa-Aesar (Karlsruhe, Germany). All spectrophotometric data were acquired using a Jasco V-530 UV-VIS spectrophotometer (Jasco International Co., Ltd., Tokyo, Japan).

### 3.3. HPLC-UV/MS Analysis of Polyphenols

#### 3.3.1. Apparatus and Chromatographic Conditions for the Analysis of Polyphenols

The identification and quantification of polyphenolic compounds was carried out using an Agilent Technologies 1100 HPLC Series system (Agilent, Santa Clara, CA, USA) equipped with G1322A degasser, G13311A binary gradient pump, column thermostat, G1313A autosampler and G1316A UV detector. The HPLC system was coupled with an Agilent 1100 mass spectrometer (LC/MSD Ion Trap SL). For the separation, a reverse-phase analytical column was employed (Zorbax SB-C18 100 × 3.0 mm i.d., 3.5 μm particle) and the work temperature was set at 48 °C. The detection of the compounds was performed on both UV and MS mode. The UV detector was set at 330 nm until 17.5 min, then at 370 nm. The MS system operated using an electrospray ion source in the negative mode. ChemStation and DataAnalysis software from Agilent were used for processing the chromatographic data. The mobile phase was a binary gradient: methanol and acetic acid 0.1% (v/v). The elution started with a linear gradient, beginning with 5% methanol and ending at 42% methanol, for 35 min; then 42% methanol for the next 3 min [34,35,36]. The flow rate was 1 mL∙min^−1^ and the injection volume was 5 µL.

The MS signal was used only for qualitative analysis based on the specific mass spectra of each polyphenol. The MS spectra obtained from a standard solution of polyphenols were integrated in a mass spectra library. Later, the MS traces/spectra of the analyzed samples were compared to spectra from library, which allows positive identification of compounds, based on spectral match. The UV trace was used for quantification of identified compounds from MS detection [34,35,36].

#### 3.3.2. Identification and Quantification of Polyphenols

Using the chromatographic conditions described above, the polyphenols eluted in less than 40 min. The detection and quantification of polyphenols was performed in UV-assisted mass spectrometry detection. Due to peak overlapping, four polyphenol-carboxylic acids (caftaric, gentisic, caffeic, and chlorogenic) were determined only based on MS spectra, whereas for the rest of the compounds the linearity of the calibration curves was very good (*R*^2^ > 0.998), with detection limits in the range of 18 to 92 ng∙mL^−1^. The detection limits were calculated as the minimal concentration yielding a reproducible peak with a signal-to-noise ratio greater than three. Quantitative determinations were performed using an external standard method; retention times were determined with a standard deviation ranging from 0.04 to 0.19 min. Calibration curves in the 0.5–50 µg∙mL^−1^ range with good linearity (*R*^2^ > 0.999) for a five point plot were used to determine the concentration of polyphenols in plant samples. For all compounds, the limit of quantification was 0.5 µg∙mL^−1^, and the limit of detection was 0.1 µg∙mL^−1^. The detection limits were calculated as minimal concentration producing a reproductive peak with a signal-to-noise ratio greater than three. The accuracy was between 94.13% and 105.3%. Accuracy was checked by spiking samples with a solution containing each polyphenol in a 10 µg∙mL^−1^ concentration. In all analyzed samples the compounds were identified by comparison of their retention times and recorded electrospray mass spectra with those of standards in the same chromatographic conditions [34,35,36].

### 3.4. Determination of Total Polyphenols and Flavonoids Content

The total phenolic content (TPC) of the extracts was measured using the Folin-Ciocalteu method with some modifications [34,37]. Two milliliters from each ethanolic extract were diluted 25 times and then mixed with Folin-Ciocalteu reagent (1 mL) and distilled water (10.0 mL) and diluted to 25.0 mL with a 290 g∙L^−1^ solution of sodium carbonate. The samples were incubated in the dark for 30 min. The absorbance was measured at 760 nm, using a JASCO UV-VIS spectrophotometer. Standard curve was prepared by using different concentrations of caffeic acid and the absorbances were measured at 760 nm. TPC values were determined using an equation obtained from the calibration curve of caffeic acid (*R*^2^ = 0.999). Total polyphenolic content was expressed as mg caffeic acid/g dry material plant (mg CAE/g plant material).

The total flavonoids content was calculated and expressed as rutin equivalents after the method described in the Romanian Pharmacopoeia (Xth Edition) [38]. Each extract (5 mL) was mixed with sodium acetate (5.0 mL, 100 g∙L^−1^), aluminum chloride (3.0 mL, 25 g∙L^−1^), and made up to 25 mL in a calibrated flask with methanol. Each solution was compared with the same mixture without reagent. The absorbance was measured at 430 nm. The total flavonoids content values were determined using an equation obtained from calibration curve of the rutin graph (*R*^2^ = 0.999).

### 3.5. In Vitro Antioxidant Activity Assays

#### 3.5.1. DPPH Bleaching Assay

The free radical scavenging activity of the ethanolic extracts was measured in terms of hydrogen donating or radical scavenging ability using this method. Trolox was chosen as a standard antioxidant. The DPPH solution (25 mM) in ethanol was prepared and 5.0 mL of this solution was added to 5.0 mL of extract solution (or standard) in ethanol at different concentrations (4–200, respectively, 5–20 µg∙mL^−1^ for extracts and standard). After 30 min of incubation at 40 °C in a thermostatic bath, the decrease in the absorbance (*n* = 3) was measured at 517 nm. The percent of DPPH discoloration was calculated as: DPPH scavenging ability = (A_control_ − A_sample_/A_control_) × 100, where A_control_ is the absorbance of DPPH radical + ethanol (containing all reagents except the sample) and A_sample_ is the absorbance of DPPH radical + sample extract. The control solution was prepared by mixing 70% vol. ethanol (5.0 mL) and DPPH radical solution (5.0 mL). Afterwards, a curve of % DPPH scavenging capacity *vs.* concentration was plotted and IC_50_ values were calculated. IC_50_ denotes the concentration of sample required to scavenge 50% of DPPH free radicals [37]. The lower the IC_50_ value is the more powerful the antioxidant capacity [37]. For all the samples, the determinations were made in triplicate and the mean values were reported.

#### 3.5.2. Trolox Equivalent Antioxidant Capacity (TEAC) Assay

In the Trolox equivalent antioxidant capacity (TEAC) assay, the antioxidant capacity is reflected in the ability of the natural extracts to decrease the color, reacting directly with the ABTS cation radical. The latter was obtained by oxidation of 2,2′-azinobis(3-ethylbenzothiazoline-6-sulfonic acid (ABTS) with potassium persulfate. Original extracts were diluted 5 times, and 3 µL from the diluted extract were added to 997 µL ABTS solution. The amount of ABTS radical consumed by the tested compound was measured at 734 nm, after 30 min of reaction time. The evaluation of the antioxidant capacity was obtained using the total change in absorbance at this wavelength; all determinations being made in triplicate [34,35].

#### 3.5.3. Ferric Reducing Antioxidant Power (FRAP) Method

FRAP method is based on the change in color of a complex with iron of the TPTZ radical, 2,4,6-tris(2-pyridyl)-s-triazine and on reduction of the ferric ion to the ferrous iron in this complex [39,40]. The color of the complex is turned from light yellowish-green to blue. This color change can be easily correlated with the antioxidant power by a spectrophotometric determination. A 2.5 mL, 10 mM TPTZ solution in 40 mM hydrochloric acid are added to 2.5 mL, 20 mM ferric chloride solution and 25 mL acetate buffer (pH 3.6). This mixture is the FRAP reagent. A 0.2 mL sample were added 0.6 mL water and 6 mL FRAP reagent. Each extract was diluted 2 to 10 mL with water. Also, a blank solution using water instead of the samples, was prepared in the same way. Trolox was used as standard antioxidant. The spectrophotometric determination was performed at 593 nm. For all the samples the determinations were made in triplicate and the mean values were reported.

#### 3.5.4. Cupric Reducing Antioxidant Capacity (CUPRAC) Assay

CUPRAC method is based on the change in color of a complex with copper of the Neocuproine, 2,9-dimethyl-1,10-phenantroline and on the reduction of copper ion (II) to the copper ion (I) from this complex. The color of the complex is turn from light green to reddish-orange. This color change can be easily correlated with the antioxidant power by a spectrophotometric determination. A 1 mL, 7.5 mM neocuproine solution, 1 mL, 10 mM copper chloride solution, and 1 mL ammonium acetate buffer at pH = 6.8 are added. This mixture is the CUPRAC reagent. A 0.1 mL sample was added to 1 mL water and 3 mL CUPRAC reagent. The mixture was incubated at room temperature for 30 min. The *Nigella* extracts were diluted 1:10 mL (*v*/*v*) with water before the analysis. Trolox, (6-hydroxy-2,5,7,8-tetramethylchroman-2-carboxylic acid), was used as standard antioxidant in a range of 10–50 µg∙mL^−1^ and the spectrophotometric determination was performed at 450 nm [31,41]. All the determinations were made in triplicate and the mean values were reported.

### 3.6. In Vivo Studies

#### 3.6.1. Animals

Experiments were performed on Wistar Bratislava male rats (*ca.* 200 g each). All animals were maintained in standard conditions and were selected by checking their diuresis with water. The experiments were realized according to the guidelines of the European Community Council Directive 86/609 and after being approved by the Ethic Committee of University of Medicine and Pharmacy ‘Iuliu Hatieganu’ from Cluj-Napoca, Romania.

#### 3.6.2. Drugs

Furosemide (Sigma-Aldrich Co.) was used as a reference diuretic drug.

#### 3.6.3. Evaluation of Diuretic Activity

The evaluation of diuretic activity test was performed on male rats as already described by Olah *et al.* and Compaore *et al*. [42,43]. Male Wistar rats were divided into four groups, of six animals each, in laboratory cages. They were fed with standard laboratory diet *ad libitum* and allowed free access to drinking water. The animals were deprived of food and water for 24 h prior to the experiment and were divided into four groups of six rats each. Then, each animal received 2.5 mL 100 g^−1^ p.o. 0.9% natrium chloride solution for hydration. After 1 h, the following substances were administrated orally.

The first group of animals, serving as control, received 1 mL/rat of distilled water, the second group received furosemide (30 mg kg^−1^, p.o.) in distilled water (as standard), and the third and fourth groups received the ethanolic extracts of *N. sativa* and *N. damascena*, respectively, at a dose of 100 mg kg^−1^ in distilled water.

#### 3.6.4. Analytical Procedure

The diuresis was expressed in mL/kg/24 h, and the diuretic index was calculated as a ratio of the values in the treated groups compared to the control group. The concentration of sodium and potassium ions were measured in urine by a potentiometric method, using a VITROS 250 Chemistry System auto-analyzer (Johnson and Johnson Clinical Diagnostic, Cluj-Napoca, Romania) and were expressed in mM/kg/24 h. Saluretic index was calculated for sodium and potassium as a ratio of their concentrations in the treated groups compared to the control group [42,43]. Uric acid concentration was determined using a colorimetric method at λ = 670 nm, through an enzymatic reaction which transforms uric acid in a colored compound, and finally was expressed in mg∙kg^−1^/24 h.

### 3.7. Statistical Analysis

The experiments were designed and the experimental data evaluated using one-way analysis of variance (ANOVA), with *p* < 0.05 as threshold for statistical significance. The statistical results confirm the hypothesis that the differences between the results are either not significant (*p* > 0.05), significant (0.001 < *p* < 0.05) or highly significant (*p* < 0.001). The average of multiple measurements (triplicates or more) was listed in the Tables together with the standard deviations. Statistical analysis was performed using Excel software package.

## 4. Conclusions

The results of the present study reveal important data regarding the phenolic composition, antioxidant and diuretic effects of seed extracts from two medicinal plants that are also considered to be important functional foods, black cumin and lady-in-a-mist. Referring to phytochemical investigations, the differences between the two species are both qualitative and quantitative. Quercetin and hyperoside were quantified only in *N. damascena* seeds extract, meanwhile, kaempferol was found only in *N. sativa*. In the hydrolyzed extracts, both quercetin and kaempferol were found in higher amounts in *N. sativa*. Nevertheless, the presence of *p-*coumaric and ferulic acids in the hydrolyzed extracts reveal the existence of several glycoside conjugates of these acids. In terms of total phenolics and flavonoids, *N. damascena* extract contains higher amounts. The antioxidant potential of the two species was tested through several electron transfer assays, which indicated *N. damascena* as exhibiting a higher free radical scavenging activity. Administration of the ethanolic extract of *N. sativa* (100 mg∙kg^−1^) resulted in a significant increase in urine volume, although less than found with the reference drug, but *N. damascena* extract did not present a diuretic effect. In reference to the elimination of Na^+^, K^+^ and uric acid, the extract of *N. sativa* showed a greater natriuretic than kaliuretic effect and a similar uricosuric effect with control and *N. damascena*. For *N. damascena*, the Na^+^/K^+^ was sub unitary, but not due to an increase of the kaliuretic effect, but mostly to a decrease of Na^+^ excretion.
